# Case Report: Exacerbation after fat grafting in patients with active localized scleroderma

**DOI:** 10.3389/fsurg.2024.1457593

**Published:** 2024-08-23

**Authors:** Shunxin Han, Juzi Liu, Feng Lu, Junrong Cai

**Affiliations:** Department of Plastic and Reconstructive Surgery, Nanfang Hospital, Southern Medical University, Guangzhou, China

**Keywords:** autologous fat grafting, morphea, localized scleroderma, active stage, pigmentary

## Abstract

**Background:**

The application of autologous fat transplantation in facial lesions of patients with localized scleroderma (LoS) has been reported in recent years.

**Objective:**

The authors report a case of worsening of active localized scleroderma after autologous fat transplantation.

**Methods:**

A man presented with neck and facial skin atrophy and pigmentation with a history of LoS. Appearing 1.5 years ago, the lesion had progressively grown in size and shape. Consent was obtained after the patient was informed of the possible surgical risks during the active phase of the disease. He underwent autologous fat grafting into the right cheek with about 30 ml Coleman fat graft.

**Results:**

Skin dyspigmentation and atrophy progressively deteriorated 1 month into therapy, with slightly increased erythema and enlargement of the lesion. Six months after the therapy, the localized scleroderma-related score worsened.

**Limitations:**

There are different factors, such as that systemic medications could affect the treatment of localized scleroderma by autologous fat transplantation. Meanwhile, considering the limitation of the 6-month follow-up period, obtaining long-term follow-up data is necessary to evaluate sustained outcomes and potential complications.

**Conclusion:**

More clinical research is needed to determine the time interval between disease inactivity and the application of any surgical procedures to avoid reactivation.

## Introduction

Local scleroderma (LoS) is a rare disease of unknown etiology without satisfactory treatment for skin sclerosis and soft-tissue atrophy. Recent studies have shown the benefits of autologous fat grafting (AFG) in the treatment of skin fibrotic disorders and soft-tissue defects (1). However, the timing of fat grafting is still controversial. Development of scleroderma includes initial inflammatory (active) and late (inactive) stages. The typically active stage is characterized by recurring patches or plaques of extensive erythema and edema, while the inactive phase is characterized by hard plaques, resulting in dermal and subcutaneous soft-tissue atrophy and skin pigmentation (2). Early intervention during the active phase of the disease can control and delay the progression of the disease. The therapeutic goal at an early inflammatory stage is to suppress disease activity (3). Adipose-derived stem cells (ASCs) in the fat graft attenuate inflammation of sclerotic skin after transplantation (4). Our previous non-randomized controlled trial showed that sequential fat grafting is safe and effective in patients with stable LoS (5). However, according to European guidelines for LoS, trauma from surgery may serve as a triggering factor for the exacerbation or reactivation of LoS, and thus should be carefully considered (6).

## Case report

**Figure 1:**
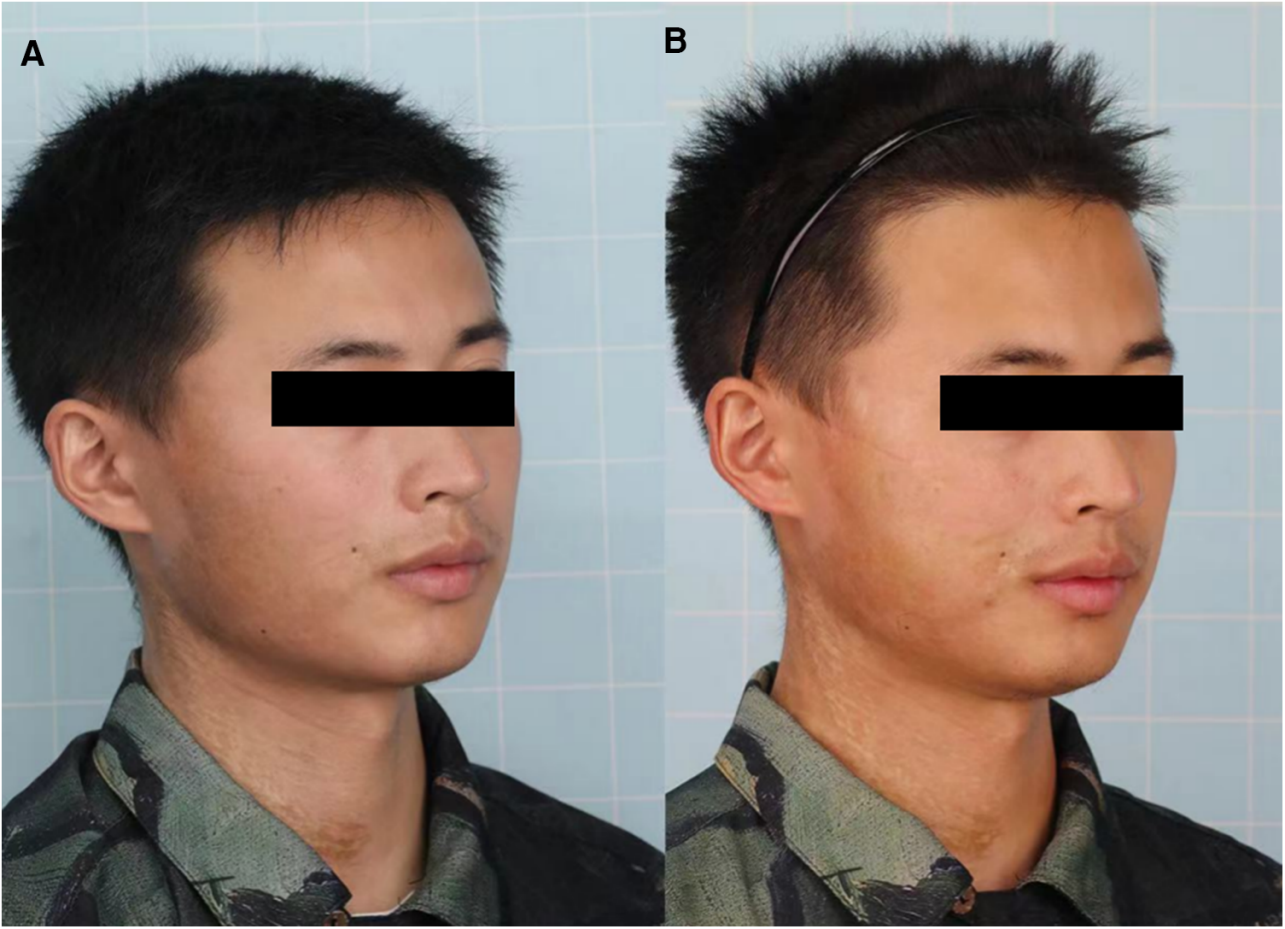
Clinical images before **(A)** and 6 months after autologous fat grafting **(B)**.

**Figure 2:**
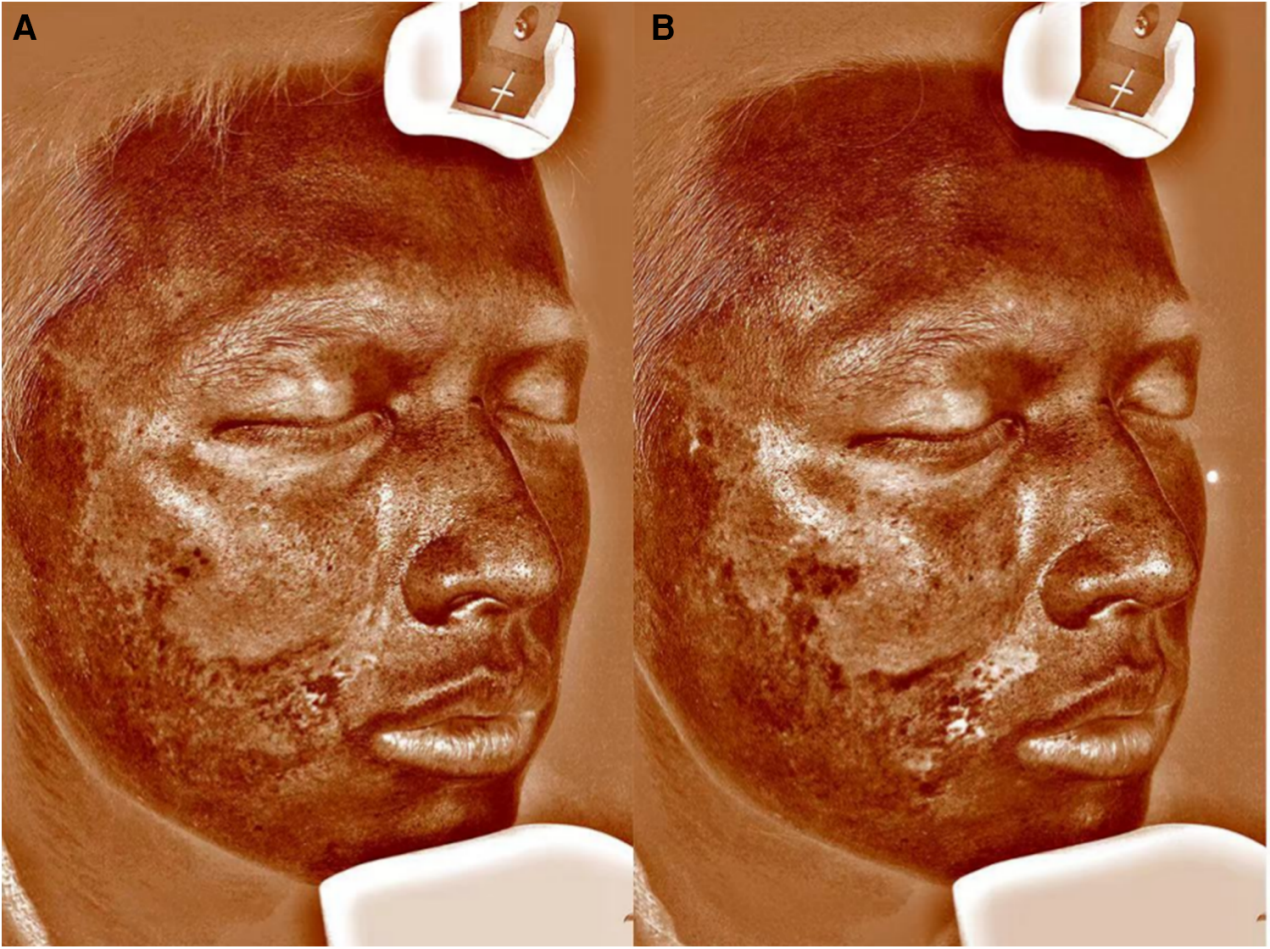
VISIA images before **(A)** and 6 months after autologous fat grafting **(B)**. VISIA skin analyzer is a facial skin analysis system.

A 25-year-old man diagnosed with LoS presented to our department for the treatment of dyspigmentation and atrophy of the skin on the right side of his cheek and neck (Figures 1A, 2A). Appearing 1.5 years earlier, the lesion had progressively grown in size with dyspigmentation, accompanied by atrophy, erythema, and stiffening of the skin. Together, these results supported a diagnosis of the active stage of LoS. We obtained consent after informing the patient of the possible risks of surgery during the active phase of the disease. A total of 30 ml lipoaspirates was injected beneath the skin lesion. After fat grating, better facial symmetry was achieved since the volume loss was recovered by fat grafts. However, skin dyspigmentation and erythema progressively deteriorated 1 month after surgery, with enlargement of the lesion. Six months after surgery, his modified Localized Scleroderma Skin Severity Index (mLoSSI) score increased from 9 to 10, and the Localized Scleroderma Skin Damage Index (LoSDI) score increased from 7 to 12, compared with before surgery (Figures 1B, 2B), which indicated disease progression. The patient didn't receive any topical treatment or systemic medication throughout.

## Discussion

Short-term hyperpigmentation is one of the few adverse events we observed in patients with active LoS receiving AFG, which spontaneously remits within 2 months. However, persisting dyspigmentation and enlarged lesion size 6 months after surgery suggest disease progression. Although LoS is usually self-limited, with frequent spontaneous regressions within 3–5 years, recurrence rates are high, even after many years of inactivity (3). In 2023, Wang and Long (7) reported a case suggesting that AFG in patients with stable scleroderma may lead to disease reignition. Disease reignition or progression could prolong the resolution time of disease activity signs and may affect the final effect of disease treatment.

In the latest European guidelines for LoS (S2k guideline) (8), it is suggested considering autologous fat stem cell transplantation for correcting soft-tissue defects in the head area in cases of linear LoS, which could be performed during the inactive or active phases of LoS and under systemic therapy (>75% consensus). It is worth noting that in the practice guidelines of diagnosis and treatment in China (9), surgery is not considered to be a cure for the disease, nor the primary method of treating the inflammatory aspects. Nevertheless surgical interventions such as fat transfer and flap reconstruction are best suited to ameliorating the sequelae of maculopathy, including visual impairment, and dysfunction in patients with inactive stage.

Moreover, topical or systemic medications may be required for the active stage of morphea. For example, the literature acknowledges PDRN could reduce disease activity and damage in quiescent localized scleroderma (10). In fact, we found no consensus on the optimal timing of intervention and/or accompanying systemic medications for AFG. Thus, after the disease has been stabilized through medication, fat grafting may offer a more effective treatment approach.

## Conclusion

The authors believe that more clinical research is needed to determine the time interval between disease inactivity and the application of any surgical procedures to avoid reactivation.

## Data Availability

The original contributions presented in the study are included in the article/Supplementary Material, further inquiries can be directed to the corresponding authors.
